# Circulating Levels of Bone Markers after Short-Term Intense Training with Increased Dairy Consumption in Adolescent Female Athletes

**DOI:** 10.3390/children8110961

**Published:** 2021-10-25

**Authors:** Panagiota Klentrou, Katherine McKee, Brandon J. McKinlay, Nigel Kurgan, Brian D. Roy, Bareket Falk

**Affiliations:** 1 Department of Kinesiology, Faculty of Applied Health Sciences, Brock University, St. Catharines, ON L2S 3A1, Canada; km15dt@brocku.ca (K.M.); nk10gw@brocku.ca (N.K.); broy@brocku.ca (B.D.R.); bfalk@brocku.ca (B.F.); 2 Centre for Bone and Muscle Health, Faculty of Applied Health Sciences, Brock University, St. Catharines, ON L2S 3A1, Canada; 3 Faculty of Applied Health and Community Studies, Sheridan College, Brampton, ON L6Y 5H9, Canada; brandon.mckinlay1@sheridancollege.ca

**Keywords:** children, exercise, Greek yogurt, bone turnover, bone metabolism, osteokines

## Abstract

Thirteen female adolescent soccer players (14.3 ± 1.3 years) participated in a cross-over, double-blind trial examining the effects of Greek yogurt (GY) consumption on bone biomarkers during 5 days of intense soccer training. The study took place over two intervention weeks, which consisted of a pre-training assessment day, 5 training days, and a post-training assessment day. Participants completed the GY condition and a carbohydrate isocaloric placebo control pudding condition (CHO) in random order, 4 weeks apart. Morning, fasted, resting blood samples were collected pre- and post-training in each condition. Total osteocalcin (tOC), undercarboxylated osteocalcin (unOC), C-terminal telopeptide of type 1 collagen (CTX), osteoprotegerin (OPG), and receptor activator nuclear factor kappa-β ligand (RANKL) were measured in serum. The results showed no effects for time (pre- to post-training) or condition, and no interaction for tOC, CTX, OPG, RANKL, and the OPG/RANKL ratio. A time-by-condition interaction (*p* = 0.011) was observed in unOC, reflecting a post-training decrease in the GY, but not the CHO condition (−26% vs. −3%, respectively). However, relative unOC (% of tOC) decreased post-training (−16%), with no differences between conditions. These findings suggest that short-term high-impact intense training had no direct catabolic impact on bone metabolism, with GY adding no benefit beyond that of the isocaloric CHO control pudding.

## 1. Introduction

Adolescence is the period of life when the highest amounts of bone mass is accrued through an increase in bone formation, without which disease and chronic issues such as osteoporosis and chronic bone mineral deficiencies can occur later in life, especially for females [1]. Although exercise, especially high-impact exercise, has been previously demonstrated to benefit bone [2,3,4,5], it is unclear what happens in adolescent females during an extended period of intense training and whether such training is beneficial or detrimental to bone formation during this crucial period of bone development. Specifically, soccer is a sport that demands high amounts of running, through training or gameplay, which is characterized by a high skeletal load shown to positively affect bone mineral content and density in male adult and adolescent players [6,7]. According to previous studies, high-impact activities also provoke exercise-induced bone responses in adolescents [3,4,8]. However, longer periods of intense exercise could potentially lead to an uncoupling or imbalance of bone turnover, favoring increased bone resorption, which may have detrimental effects on bone mass and health [9].

Furthermore, calcium is an important nutrient when it comes to bone development and accretion during the pubertal years [10,11]. However, studies have indicated that only about 70% of the adolescent population is consuming the recommended daily allowance (RDA) of dietary calcium intake (1300 mg), particularly in adolescent females [1,12,13]. Since calcium requirements increase during periods of rapid growth, low dietary calcium can result in low bone mineral density and content, leading to increased risk of fractures [14,15]. Thus, calcium consumption, whether lower or higher than the recommended intake, could exacerbate or counterbalance, respectively, any potentially negative effects of intense exercise on bone [15]. In addition to calcium, protein supplementation may also play an instrumental role in bone tissues that undergo stress during exercise and attenuate some of the negative effects of overtraining [16]. For example, according to a recent study in adult male endurance athletes, a protein beverage combined with carbohydrates consumed immediately post-exercise resulted in increased circulating levels of bone formation markers, and decreased levels of bone resorption markers [17]. However, the protein requirements for child and adolescent athletes or highly active youth are still unspecified due to the lack of studies examining the effects of protein consumption on bone turnover in young athletes, who may have higher needs for protein than non-athletic youth. 

Greek yogurt (GY) has both a higher protein and calcium content, nearly triple and double that of regular yogurt, respectively [18,19,20,21]. Due to these relatively higher concentrations, GY may serve as a practical option to increase total calcium and protein intake, yielding beneficial bone adaptations. Providing effective nutrition to adolescent athletes, specifically females, may counter any potential negative effects during periods of intense exercise training on bone. Therefore, this study was designed to investigate the effects of GY consumption on bone markers of bone turnover during short-term intense training in adolescent female soccer athletes. Specifically, we examined whether consumption of three daily doses (at breakfast, immediately following each training workout, and before bedtime) of GY in comparison to an isocaloric carbohydrate control pudding (CHO, designed for the study) would affect bone metabolic markers during a 5-day high volume, high intensity training camp in female soccer players. Bone markers reflect the bone formation and resorption processes in the body and can give a dynamic measure of these processes as they are stimulated by biomechanical forces—such as exercise and training [22]. The markers of bone turnover measured in this study included total osteocalcin (tOC), undercarboxylated osteocalcin (unOC), and C-terminal telopeptide of type 1 collagen (CTX). Osteoprotegrin (OPG) and receptor activator nuclear factor kappa-β ligand (RANKL) were also measured because they reflect the relative balance of bone turnover and can provide insight into which process (resorption or formation) is favoured [23]. It was hypothesized that during the period of short-term intense training, the consumption of GY would help maintain the systemic levels of tOC, unOC, CTX, OPG, RANKL, and the OPG/RANKL ratio near their pre-training levels, compared with the isocaloric carbohydrate control pudding, where we expected to see a catabolic effect of intense training, reflected by an increase in unOC, CTX, and/or RANKL, with unchanged or decreased tOc and/or OPG.

## 2. Materials and Methods

### 2.1. Participants

A total of 21 competitive, female soccer players (12–16 years), were recruited from local soccer clubs in Southern Ontario, Canada to participate in a clinical trial on the effects of GY consumption on indices of inflammation and recovery [24]. Participants had a minimum 2 years of elite competitive experience, training ≥ 3 sessions/week, were free of any musculoskeletal injury or medical condition that would prevent them from participating in maximal exercise, had no hypersensitivity or allergy to dairy products, and did not take any medication or supplements. All participants and their parents/guardians received a thorough explanation of the study’s purpose, procedures, benefits, and potential risks, and consent was obtained from both the participants and their parents/guardians prior to study commencement. The study was cleared by the Research Ethics Board of Brock University and was registered at Clinicaltrials.gov (NCT03947801).

Of the 21 recruited participants, 13 completed all parts of the study while the other 7 dropped out for reasons not pertaining to the study. The mean age of the 13 completed participants was 14.3 ± 1.3 years, with no differences in their anthropometric characteristics between intervention weeks (Table 1). Participants were 0.8 ± 0.8 years from their estimated age of peak height velocity, and post-menarcheal, with only mild irregularities reported.

### 2.2. Study Design

**Figure 1:**
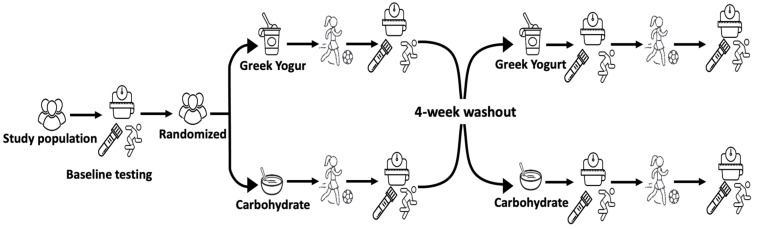
Study design.

The study was carried out using a cross-over, randomized, double-blind, placebo-controlled design [24]. All participants participated in two intervention weeks, each consisting of a pre-training testing day, 5 days of consecutive soccer training, and one post-training testing day. Intervention weeks were scheduled in a random order and 4 weeks apart to correspond with the same phase of menstrual cycle for each player and to allow for an adequate wash-out period. Following the first week of intervention, participants resumed regular soccer activities in their respective teams for four weeks (wash-out period). Upon completion of the wash-out period, participants were again reminded to not partake in any physical activity 24 h prior to baseline testing. The second week of intervention was identical to the first, with the exception that supplementation was crossed over (i.e., each participant received the opposite supplement from the first week of training). An overview of the cross-over design can be found in Figure 1.

### 2.3. Experimental Procedures and Measurements

During the training camps, participants were tested twice: on the day before (pre) and the day following (post) the 5-day soccer-specific training. Participants arrived at the University for pre- and post-training testing at 08:00 AM in Canada. Participants refrained from exercise 24 h prior to the initial baseline data collection visit. Standing and seated height were measured using a portable stadiometer (SECA-217, Canada), and recorded to the nearest 0.5 cm. These measurements were used to calculate somatic maturity offset (years from the age of peak height velocity), as previously described [25]. Body mass and percent body fat (%BF) were measured by bioelectrical impedance analysis (Biospace.228, Los Angeles, CA, USA), and were recorded to the nearest 0.1 kg and 0.1%, respectively. A fasted, resting venous blood sample was then taken, followed by a standardized breakfast, which included one granola bar, one muffin, fruit (banana, apple, and strawberries) and a juice box or water (~400–500 kcal total). Participants were instructed to eat the same breakfast during the pre- and post-training testing sessions. They were then provided with a food frequency questionnaire (Block 2014.1_6Mo, Nutrition Quest, Los Angeles, CA, USA), to be completed by the end of the study. During the period between the first blood draw and the first training session, participants were randomized to one of two experimental conditions—Greek yogurt (GY) or an isocaloric study-designed carbohydrate pudding (CHO)—by an independent research assistant.

### 2.4. Training Sessions

Training sessions began the day following baseline blood draw and consisted of 5 consecutive days of soccer-specific training, structured to mimic a heavy-volume, high-intensity training week (microcycle). Training sessions occurred at 18:00–20:00 h each day and were administered by a certified technical soccer coach and knowledgeable training staff. The coach-to-participant ratio during all training sessions was 1:3.

Each session began with a 15-min dynamic warm-up followed by 90-min of soccer-specific training, ending with a 15-min cool-down. The 90-min of soccer-specific drills were performed at maximal effort and consisted of agility, sprinting and plyometric drills as well as ball-handling, small-sided games (rondo), and shooting. The work-to-rest ratio during activities and drills was 1:1 and 1:2, while it was increased up to 1:3 for more explosive drills (i.e., plyometric exercise). The intensity of the training was self-rated by participants using a standard rating of perceived exertion scale, and the mean rating was similar between the two intervention conditions.

### 2.5. Nutritional Intervention

Participants consumed three servings of 160 g of GY (~115 Kcals, 17 g protein, ~11.5 carbs) (Skotidakis Inc., St. Eugene, ON, Canada) [26], or 30 g of isocaloric CHO pudding (~115 Kcal, 0.04 g protein, ~28.6 g carbs) immediately following the training session, 1 h prior to bedtime, as well as one serving between breakfast and lunch on the subsequent day. Following the last training session, participants consumed only two servings (immediately post-session and prior to bedtime). The study-designed pudding was created daily in the laboratory using a combination of fat-free vanilla Jell-O instant pudding (6 g) and maltodextrin (24 g) mixed in water. Both supplements were served in clear containers by an independent research assistant, separate from the coaching staff (double-blind). While the palatability and taste of GY was noticeable to participants, the true contents of the study designed pudding were concealed. Participants self-reported 100% compliance in supplement consumption during both intervention conditions.

Although the literature reports a weak-to-moderate effect on timing of protein consumption and bone metabolism immediately post-exercise [17], we chose to give participants protein immediately post-exercise to partly control supplement consumption adherence and to mimic the design of similar studies using milk for comparison purposes. Due to the specific digestion and absorption kinetics of casein protein (which comprises most of the protein in GY), we gave participants 160 g of GY prior to sleep, as research shows that protein, specifically casein, prior to sleep may maintain an elevated net protein balance throughout sleep [27]. In addition to protein timing, we also wanted to ensure that our supplementation of GY allowed participants to meet the overall protein intake of 1.6 g/kg/day in efforts to maximize bone adaptations.

During both training portions of the study, participants were asked to self-report all food consumed in a 24 h period for 5 consecutive days. The 24 h food recall form was provided to each participant in a folder with a portion sheet stapled to the inner flap. Each day, participants would complete and return the food record and be provided with a blank one for the subsequent 24 h period. Photocopies of the food records over the course of the first training week were made available to participants prior to the second week of training. Participants were instructed to follow the same diet as best they could. All food records were analyzed using a diet analysis program (Food Processor, ESHA Inc., Salem, OR, USA), and inputted and analyzed by the same examiner for consistency. Habitual dietary intake was assessed using the Block Food Frequency Questionnaire (FFQ), designed to assess dietary habits through a recall of foods eaten in the last 6 months (Berkly, CA, USA).

### 2.6. Blood Collection and Analysis

Blood samples were obtained on four occasions (pre- and post-training during training week 1 and 2) between the hours of 08:00 and 10:00 AM in Canada after an overnight fast of 10–12 h. On each occasion, a total of 10 mL of blood was collected from an antecubital vein by a certified independent phlebotomist using a standard venipuncture technique. Blood was collected into SST vacutainer tubes and was allowed to clot for 20 min at room temperature (23 °C) before being centrifuged at 4 °C for 15 min at 1405 RCF (g). Serum was separated and aliquoted into 0.5 mL polyethylene cryotubes that were stored at −80 °C until analysis.

All analytes were measured in serum, in duplicate, and the average coefficients of variations (CV) were estimated in-house. tOC and OPG were measured using a microbead multiplex kit (Human Bone Magnetic Bead Panel, cat.# HBNMAG-51K-08, EMD Millipore, Darmstadt, Germany). The average inter- and intra-assay coefficients of variation (CV) for tOC were 2.7% and 8.4%, and for OPG were 0.1% and 1.5%, respectively. unOC was measured using an ELISA kit (cat. # MK118, Takara Bio, San Jose, CA, USA) with an inter-assay CV of 4.7% and an intra-assay CV of 7.0%. CTX was measured using an ELISA kit (cat. # E-EL-H0835, Elabscience, Wuhan, China) with an inter-assay CV of 7.0% and an intra-assay CV of 5.8%. RANKL was measured using a microbead multiplex kit (Human RANKL MAG Bead Single Plex Kit, cat.# HRNKLMAG-51K-01, EMD Millipore, Darmstadt, Germany) with an average intra-assay CV of 5.2%, and an inter-assay CV of 5.7%. The OPG:RANKL ratio was calculated for each participant at each time point by dividing the OPG concentration (in pg/mL) with the RANKL concentration (in pg/mL).

Although each participant was in the same phase of their menstrual cycle for the two training weeks, estradiol concentrations were measured during the pre-training visits only to confirm that each participant begun the intervention conditions with similar estradiol levels. Specifically, estradiol was measured in serum using an ELISA assay (Human Estradiol E2 kit, Abcam, Toronto, ON, Canada). The estradiol inter-assay CV was 5.2%, and the intra-assay CV was 8.0%. The sensitivity of this assay was 10–1000 pg/mL with 101.3% recovery in serum.

### 2.7. Statistical Analysis

Prior to analysis, data were screened for normality using the Shapiro–Wilk test, z-scores for skewness and kurtosis of ±3, and visual screening of histograms for symmetry. According to the screening, tOC, unOC, CTX, RANKL, and the OPG/RANKL ratio were not normally distributed and were log-transformed for the analysis. Three participants with missing values for unOC, CTX, and RANKL due to undetectable concentrations (i.e., below the detection limit of the biochemical assay) were excluded from the analysis of these markers.

Paired *t*-tests were used to examine differences between the two pre-training visits in terms of physical characteristics, training history, and estradiol concentration. Differences between conditions and changes over time for each of the biomarkers were examined using a two-way repeated measures analysis of variance, with two within-subject main-effects (time and condition). An alpha value of *p* < 0.05 was used to determine statistical significance. Statistical analyses were performed using SPSS version 25.0 for Windows.

## 3. Results

As per the study design, participants consumed significantly more protein in the GY condition compared to the CHO condition (*p* ≤ 0.001), and significantly more carbohydrates in the CHO condition compared to the GY condition (*p* ≤ 0.001), with no differences in daily energy or fat intake between GY and CHO (Table 2). Likewise, when the training diet (via food record) was compared to participants’ habitual diets (via FFQ), relative habitual protein and carbohydrate consumption were significantly lower when compared to the intervention conditions (Table 2), where GY (*p* ≤ 0.001) and CHO (*p* = 0.003) were consumed, respectively, with no differences with respect to the daily energy intake (*p* = 0.22) and relative fat consumption (*p* = 0.82).

Basal estradiol concentrations were similar at the beginning of the GY and CHO intervention conditions (13.1 ± 11.2 pg·mL^−1^ versus 13.4 ± 12.1 pg·mL^−1^, respectively). Resting, pre-training concentrations of bone turnover markers and osteokines were not significantly different between conditions, but there was high variability for all biomarkers, with the %CV ranging from 19% in OPG to 88% in the OPG/RANKL ratio (Table 3). Moreover, tOC showed no main effect for condition (F = 0.021; *p* = 0.886, partial η^2^ = 0.001) or time (F = 0.285; *p* = 0.589, partial η^2^ = 0.012), and no interaction (F = 0.280; *p* = 0.602, partial η^2^ = 0.012) (Table 3). In contrast, unOC showed no condition effect (F = 0.15; *p* = 0.707, partial η^2^ = 0.008), but there was a significant main effect for time (F = 16.0; *p* = 0.001, partial η^2^ = 0.471) and a significant time-by-condition interaction (F = 7.919; *p* = 0.011, partial η^2^ = 0.306), reflecting a −26% decrease from pre- to post-training in the GY condition (95% CI = −14% to −38%; *p* = 0.002), which was only −3% (95% CI = −17% to +10%; *p* = 0.638) in the CHO condition (Table 3). However, the relative unOC to tOC (% of OC) ratio showed a significant main effect for time (F = 12.2; *p* = 0.003, partial η^2^ = 0.405), reflecting an overall −16% decrease (95% CI = −28% to −4%; %; *p* = 0.011) from pre- to post-training, but no significant time-by-condition interaction (F = 2.80; *p* = 0.0112, partial η^2^ = 0.135) and no condition effect (F = 0.002; *p* = 0.961, partial η^2^ = 0.000). There was no main effect for condition (F = 0.137; *p* = 0.715, partial η^2^ = 0.006) or time (F = 0.060; *p* = 0.809, partial η^2^ = 0.002), and no interaction (F = 0.064; *p* = 0.802, partial η^2^ = 0.003) for CTX (Table 3). Likewise, OPG showed no effect for condition (F = 0.35; *p* = 0.56, partial η^2^ = 0.014) or time (F = 0.30; *p* = 0.59, partial η^2^ = 0.012), and no interaction (F = 1.65; *p* = 0.21, partial η^2^ = 0.064). RANKL also showed no effect for condition (F = 0.11; *p* = 0.74, partial η^2^ = 0.005) or time (F = 0.48; *p* = 0.49, partial η^2^ = 0.019), and no interaction (F = 2.73; *p* = 0.11, partial η^2^ = 0.102). Finally, for the OPG/RANKL ratio, there was no effect for condition (F = 0.16; *p* = 0.69, partial η^2^ = 0.007) or time (F = 0.13; *p* = 0.73, partial η^2^ = 0.005), and no significant interaction (F = 1.22; *p* = 0.28, partial η^2^ = 0.049) (Table 3).

## 4. Discussion

This study provides new evidence on the effects of consuming GY on bone biomarkers following 5 days of intense training in adolescent girls. We found no training-induced adverse effects on tOC, CTX, OPG, RANKL, and their ratio in either the GY or the isocaloric carbohydrate control condition. unOC decreased significantly at the end of the intense training period in the GY condition, but not in the CHO condition. However, relative unOC, expressed as a percentage of tOC, was reduced post-training in both the GY and CHO conditions, which may reflect lower bone resorption.

Contrary to previous reports in adult females [9], we did not observe a catabolic effect of training. This may be because our participants did not appear to be in a negative energy balance while the young adult females in the Ihle and Loucks study performed 5 consecutive days of exercise at 70% $\dot{V}$O_2_ max in an energy-restricted state [9]. Additionally, adolescence is a crucial period of high bone turnover, and although this study had the adolescent girls perform high-volume, intense training over 5 consecutive days, there is a possibility that this period of high turnover in bone protects them from the deficits of overtraining or chronic intense exercise. In other words, it might be possible that their growing processes are able to counter any negative effects of high-impact intense training for multiple days, especially when combined with good energy and nutritional intake.

Since there is limited comparable research available in the pediatric population, acceptable baseline ranges for bone turnover markers are currently unknown. However, the tOC concentrations in our adolescent soccer players were very similar to those previously reported in adolescent female athletes (ages 14–18 years), i.e., within a 6000 to 8000 pg·mL^−1^ range [28]. In circulation, tOC is considered a marker of bone formation, yet its exact role in the control of bone matrix formation, mineralization, or maintenance is not fully understood [29,30]. It is generally accepted, however, that circulating tOC levels are indicative of osteoblast activity, and they were found to increase in adults during exercise [31]. In children, a study comparing the response to plyometric exercise in normal-weight and overweight adolescent females found that tOC levels increased significantly 1 h post-exercise session [32]. Their results suggest that using a higher impact modality of exercise may be beneficial for promoting positive bone turnover responses during this critical period for bone accrual (i.e., adolescence). Thus, our results of no change in bone markers over 5 days of intense high impact exercise training may also suggest that the increased mechanical stimulus of the activity could counterbalance the potential negative effect of the high intensity, repetitive load, at least in the short term (one week).

Circulating osteocalcin comprises both undercarboxylated and carboxylated forms [30]. Of the total amount of osteocalcin that is released into the circulation, a substantial proportion (40–60%) is unOC [30]. A higher percentage of unOC has been associated with an increased risk of bone fracture in older adults, particularly women. Therefore, the detection of circulating unOC has long been recognized as having clinical predictive value as a biomarker and indicator of fracture risk [30]. In humans, however, most bone studies have measured total osteocalcin instead of the undercarboxylated form. Interestingly, we found a training-induced decrease in unOC in the GY condition, but not in the CHO condition, which could be interpreted as GY having preserved bone turnover after training better than CHO. However, this can be misleading because unOC is implicated in glucose metabolism, so this decrease could reflect differences in the overall metabolic activity between conditions. The results of the few studies that have measured unOC in response to exercise have been equivocal [33,34]. Since unOC has been reported to regulate glucose metabolism, which provides energy to muscles during exercise, it has been suggested that skeletal muscle and adipose tissue respond to osteocalcin by increasing their sensitivity to insulin [35,36,37]. It is possible, therefore, that unOC concentrations were positively impacted in both of our nutritional conditions by the added 345 kcal·d^−1^ (3 servings × 115 kcals per serving). This could also counterbalance any training-induced catabolic effect on bone formation as energy availability does play a role in bone metabolism [31]. Indeed, relative unOC, which is a better reflection of bone turnover status than unOC alone, decreased following the intense training in our adolescent athletes with no differences between GY and CHO. This finding supports the suggestion that there was no negative effect on bone turnover following intense training in our adolescent athletes.

The resting CTX levels seen in this study were lower than previously measured in young adult women [38], as well as in adolescent swimmers [39]. These low levels of CTX could be due to either the younger age of our adolescent female participants or their involvement in a high impact sport. Since this is the period of peak bone formation in their lives, resorption levels may be lower in adolescent females, and even lower in these participants as they are high-impact athletes. Importantly, we found no significant changes in CTX following 5 consecutive days of intense high-impact training, with consumption of both GY and CHO (3 servings per day), in this unique cohort of highly active, elite adolescent female athletes. Previously, protein combined with carbohydrate consumption was shown to acutely decrease CTX concentrations 2–4 h after exhaustive running in endurance-trained male adults [17]. Additionally, a study by Bridge et al. [40], examining the effect of GY supplementation over a 12-week period of training, found that CTX increased acutely after exercise, but returned to baseline over time in the group that consumed GY. However, the results of both of these studies were in adult males and cannot be directly compared with our findings in adolescent females. There are no studies on GY consumption in adolescent females. However, our group previously investigated the impact of 12 weeks of dairy consumption and exercise on bone-biomarkers in female adolescents with increased adiposity, who were randomly assigned to either a group with dairy consumption at the recommended amount or a lower dairy consumption group [41]. We found that CTX decreased after 12 weeks of moderate intensity exercise intervention, irrespective of dairy consumption [41]. Similarly, Kurgan et al. [32] also reported a significant acute decrease in CTX following plyometric exercise in both normal weight and overweight adolescent females. The decrease in CTX was seen immediately post-exercise as well as 1 h post-exercise, suggesting a shift towards a supressed bone resorption, i.e., an anabolic response to high-impact exercise in female adolescents. The present study demonstrated that CTX levels were kept constant after 5 consecutive days of high-impact exercise, suggesting that the acute decreases in CTX following a single exercise session are transient in this young population, and that the 5 days of intense training was not long enough to alter bone turnover in this young population.

OPG levels did not change during the training weeks, and GY provided no added benefit compared with CHO. OPG is secreted by osteoblasts and osteogenic stromal stem cells to protect the skeleton from excessive bone resorption by binding to RANKL and preventing the interaction with RANK [23]. In our study, both RANKL and the OPG/RANKL ratio were not affected by the intense training, suggesting no increase in catabolism due to training. A study in college females (non-regular exercisers) also found no significant changes in the OPG and RANKL levels after a 12-week combined endurance and resistance training program [42]. As RANKL is an indicator of osteoclast activity, our research potentially demonstrates that intense training does not increase osteoclast activity in young female athletes. Since soccer is a high-impact activity, these athletes already have a positive bone formation to resorption ratio.

This study has several strengths including its cross-over design and ecological validity, which makes its results very compelling. We opted to have participants complete 5 consecutive days of soccer-specific training sessions, as increased training frequency is common in this population. We chose a 5-day training intervention as this is a typical duration for high level soccer players when leading into a game or tournament, so we tried to mimic that training. Another strength of the study is that all measurements were performed in the morning and before breakfast, thus eliminating diurnal fluctuations and nutritional effects. This was important since most bone turnover markers are stable and do not vary much throughout the day or when food is consumed, but CTX is known to be at its peak during morning hours and is influenced by food intake [43]. The main limitation of this study was its small sample size (*n* = 13). Although this sample size is similar to previous cross-over studies, the study was underpowered (observed power 1 − β = 0.10 to 0.60). Originally, the clinical trial was designed with a calculated sample size of *n* = 20, which was required to reach a power of (1 − β) = 0.8, a large effect size (i.e., partial η^2^ = 0.14), and *p* = 0.05. However, of the 21 participants initially enrolled in the study, only 13 completed both intervention conditions. Another limitation is that bone turnover markers reflect overall bone homeostasis, i.e., the activity of osteoblasts and osteoclasts in physiological conditions, and do not reflect bone cellular activity at a specific site [44]. Since bone biopsy in humans is not possible, mechanistic studies should be conducted in animal models to confirm these findings. Alternatively, future studies should examine the influence of longer-term intense exercise training and dietary interventions on bone markers and bone mineral content in specific skeletal sites among pediatric populations to see whether these findings persist/change over time, with interventions designed to protect peak bone mass acquisition in active youth.

## 5. Conclusions

When compared to an isocaloric carbohydrate control pudding, three daily servings of GY during high-intensity, high-impact, short-term training, did not affect bone formation or resorption markers, either at rest or in response to training. We also found no markable adverse effects on markers of bone metabolism because of the intense soccer training. This brings us to the conclusion that, overall, when female adolescent athletes are well nourished, they are protected from the potential negative effect on bone metabolism, and eventually bone accrual, due to high volume training, at least in the short-term. Promoting bone turnover in favour of bone formation during adolescence is beneficial because it can promote bone accretion, leading to higher peak bone mass and improved bone mineral density in the long term.

## Figures and Tables

**Table 1:** Physical characteristics of the female soccer players (N = 13) at pre-training during each nutritional intervention. Values are mean ± standard deviation.

	Greek Yogurt	Carbohydrate
Height (cm)	165.9 ± 5.2	166.0 ± 5.3
Body mass (kg)	59.1 ± 7.5	59.3 ± 7.4
Body fat (%)	22.2 ± 6.5	22.2 ± 5.4

**Table 2:** Habitual nutrition intake, and intervention nutritional intake of the adolescent female soccer players (N = 13). Values are mean ± standard deviation.

Condition	Energy (kcal)	Fat (g·kg^−1^·d^−1^)	Carbohydrate (g·kg^−1^·d^−1^)	Protein (g·kg^−1^·d^−1^)
Habitual	1622 ± 502	1.1 ± 0.4	3.3 ± 0.7	1.1 ± 0.3
GY condition	1892 ± 287	1.0 ± 0.2	4.0 ± 1.0	1.9 ± 0.3 *
CHO condition	1959 ± 441	1.0 ± 0.4	5.2 ± 1.2 †	1.0 ± 0.3

Habitual diet was assessed by food frequency questionnaire; energy and macronutrient consumption (including the supplements) during the Greek yogurt (GY) and isocaloric control (CHO) conditions were assessed using diet records. * indicates GY significantly greater (*p* < 0.05) than CHO and habitual. † indicates CHO significantly greater (*p* < 0.05) than GY and habitual.

**Table 3:** Resting, morning concentrations of bone turnover markers and osteokines during each intervention condition in female adolescent soccer players.

Marker	Group	Pre-Training	Post-Training
tOC (ng·mL^−1^)	GY	74.0 ± 29.1 (39%)	74.0 ± 29.9 (40%)
	CHO	73.2 ± 30.2 (41%)	78.0 ± 33.5 (43%)
unOC (ng·mL^−1^) *^,^#	GY	8.9 ± 4.5 (50%)	6.6 ± 3.5 (54%)
	CHO	8.6 ± 4.5 (52%)	8.4 ± 4.6 (54%)
unOC/tOC (%) *	GY	12.4 ± 6.1 (49%)	9.4 ± 5.0 (53%)
	CHO	11.6 ± 4.6 (40%)	10.5 ± 4.4 (42%)
CTX (pg·mL^−1^)	GY	0.17 ± 0.11 (65%)	0.16 ± 0.10 (62%)
	CHO	0.16 ± 0.11 (68%)	0.16 ± 0.11 (68%)
OPG (pg·mL^−1^)	GY	1388.2 ± 475.9 (34%)	1223.8 ± 233.0 (19%)
	CHO	1206.8 ± 363.4 (30%)	1273.1 ± 344.9 (27%)
RANKL (pg·mL^−1^)	GY	34.3 ± 22.1 (64%)	29.8 ± 21.4 (72%)
	CHO	30.3 ± 21.4 (71%)	35.0 ± 17.9 (51%)
OPG/RANKL (ratio)	GY	57.4 ± 48.5 (84%)	69.5 ± 57.1 (82%)
	CHO	57.1 ± 48.2 (84%)	50.6 ± 44.7 (88%)

Values are mean ± standard deviation (% coefficient of variation); t-OC= total osteocalcin (N = 13); unOC = undercarboxylated osteocalcin (N = 10); unOC/tOC = relative undercarboxylated osteocalcin to total osteocalcin (N = 10); CTX = C-terminal telopeptide of type I collagen (N = 10); OPG = osteoprotegerin (N = 13); RANKL = receptor activator nuclear factor kappa-β ligand (N = 10); OPG/RANKL ratio (N = 10); * denotes significant main effect for time; # denotes significant time by condition interaction.

## Data Availability

The datasets used and/or analysed during the current study are available from the corresponding author on reasonable request. The data are not publicly available due to REB restrictions.
