# Correction: Klentrou et al. Circulating Levels of Bone Markers after Short-Term Intense Training with Increased Dairy Consumption in Adolescent Female Athletes. *Children* 2021, *8*, 961

**DOI:** 10.3390/children10010096

**Published:** 2023-01-03

**Authors:** Panagiota Klentrou, Katherine McKee, Brandon J. McKinlay, Nigel Kurgan, Brian D. Roy, Bareket Falk

**Affiliations:** 1 Department of Kinesiology, Faculty of Applied Health Sciences, Brock University, St. Catharines, ON L2S 3A1, Canada; 2 Centre for Bone and Muscle Health, Faculty of Applied Health Sciences, Brock University, St. Catharines, ON L2S 3A1, Canada; 3 Faculty of Applied Health and Community Studies, Sheridan College, Brampton, ON L6Y 5H9, Canada

## Text Correction

There was an error in the original publication [1]. The somatic maturity estimation was mistakenly based on the male equations. Using the appropriate female-specific equations suggested by Mirwald et al. [2], participants were 2.4 ± 0.7 years from their estimated age of peak height velocity.

A correction has been made to **2. Materials and Methods**, *2.1. Participants*, Paragraph 2 as follows:

“Of the 21 recruited participants, 13 completed all parts of the study, while the other 7 dropped out for reasons not pertaining to the study. The mean age of the 13 completed participants was 14.3 ± 1.3 years, with no differences in their anthropometric characteristics between intervention weeks (Table 1). Participants were 2.4 ± 0.7 years from their estimated age of peak height velocity, and post-menarcheal, with only mild irregularities reported”.

## Error in Table

In the original publication, there was a mistake in Table 3 as published. The units for CTX in Table 3 should be ng·mL^−1^. The corrected Table 3 appears below.

The authors state that the scientific conclusions are unaffected. This correction was approved by the Academic Editor. The original publication has also been updated.

## Figures and Tables

**Table 3:** Resting, morning concentrations of bone turnover markers and osteokines during each intervention condition in female adolescent soccer players.

Marker	Group	Pre-Training	Post-Training
tOC (ng·mL^−1^)	GY	74.0 ± 29.1 (39%)	74.0 ± 29.9 (40%)
	CHO	73.2 ± 30.2 (41%)	78.0 ± 33.5 (43%)
unOC (ng·mL^−1^) *^,#^	GY	8.9 ± 4.5 (50%)	6.6 ± 3.5 (54%)
	CHO	8.6 ± 4.5 (52%)	8.4 ± 4.6 (54%)
unOC/tOC (%) *	GY	12.4 ± 6.1 (49%)	9.4 ± 5.0 (53%)
	CHO	11.6 ± 4.6 (40%)	10.5 ± 4.4 (42%)
CTX (ng·mL^−1^)	GY	0.17 ± 0.11 (65%)	0.16 ± 0.10 (62%)
	CHO	0.16 ± 0.11 (68%)	0.16 ± 0.11 (68%)
OPG (pg·mL^−1^)	GY	1388.2 ± 475.9 (34%)	1223.8 ± 233.0 (19%)
	CHO	1206.8 ± 363.4 (30%)	1273.1 ± 344.9 (27%)
RANKL (pg·mL^−1^)	GY	34.3 ± 22.1 (64%)	29.8 ± 21.4 (72%)
	CHO	30.3 ± 21.4 (71%)	35.0 ± 17.9 (51%)
OPG/RANKL (ratio)	GY	57.4 ± 48.5 (84%)	69.5 ± 57.1 (82%)
	CHO	57.1 ± 48.2 (84%)	50.6 ± 44.7 (88%)

Values are mean ± standard deviation (% coefficient of variation); t-OC = total osteocalcin (N = 13); unOC = undercarboxylated osteocalcin (N = 10); unOC/tOC = relative undercarboxylated osteocalcin to total osteocalcin (N = 10); CTX = C-terminal telopeptide of type I collagen (N = 10); OPG = osteoprotegerin (N = 13); RANKL = receptor activator nuclear factor kappa-β ligand (N = 10); OPG/RANKL ratio (N = 10); * denotes significant main effect for time; # denotes significant time by condition interaction.
